# Effect of Dysphagia Rehabilitation Using Kinesiology Taping on Oropharyngeal Muscle Hypertrophy in Post-Stroke Patients: A Double Blind Randomized Placebo-Controlled Trial

**DOI:** 10.3390/healthcare8040411

**Published:** 2020-10-19

**Authors:** Young-Jin Jung, Hee-Jeong Kim, Jong-Bae Choi, Ji-Su Park, Na-Kyoung Hwang

**Affiliations:** 1 Department of Radiological Science at Health Sciences Division, DongSeo University, 47 Jurye-ro, Sasang-gu, Busan 47011, Korea; microbme@dongseo.ac.kr; 2 Department of Occuptional Therapy, Kyungdong University, 815, Gyeonhwon-ro, Munmak-eup, Wonju, Gangwon-do 26495, Korea; hjk@kduniv.ac.kr; 3 Department of Occupational Therapy, Sangji University, 83, Sangjidae-gil, Wonju, Gangwon-do 26339, Korea; cjb3798@naver.com; 4 Advanced Human Resource Development Project Group for Health Care in Aging Friendly Industry, Dongseo University, 47 Jurye-ro, Sasang-gu, Busan 47011, Korea; 5 Department of Occupational Therapy, Seoul North Municipal Hospital, 38 Yangwonyeokro, Jungnang-gu, Seoul 02062, Korea

**Keywords:** dysphagia, kinesiology taping, rehabilitation, suprahyoid muscles, tongue muscle, ultrasound

## Abstract

Background: It has recently been shown that suprahyoid muscle exercise using kinesiology taping (KT) increases the activation of the suprahyoid muscle in healthy adults, suggesting a potential therapeutic clinical exercise for dysphagia rehabilitation. This study investigated the effect of dysphagia rehabilitation using KT in stroke patients with dysphagia. Methods: Thirty subjects in South Korea were enrolled in this prospective placebo-controlled double-blind study. Participants were randomly assigned to the experimental and sham groups. In the experimental group, the tape was attached to the hyolaryngeal complex, pulled downward with approximately 70% tension, and then attached to the sternum and the clavicle bilaterally. In the sham group, the tape was applied similarly but without the tension. Both groups performed voluntary swallowing 50 times (10 times swallowing per set, times 5 sets) a day for 4 weeks with KT applied. Outcome measures were assessed using portable ultrasound equipment. The parameter measured was the change in thickness of the tongue muscle, mylohyoid muscle, and the anterior belly of the digastric muscle. Results: The experimental group showed statistically significant changes in the thickness of the tongue muscle, mylohyoid muscle, and anterior belly of the digastric muscle than the sham group (*p* = 0.007, 0.002, and 0.001). Conclusion: Dysphagia rehabilitation using KT is a technique that may promote oropharyngeal muscle thickness in patients with dysphagia after stroke.

## 1. Introduction

Dysphagia has a prevalence of about 50% up to 80% in stroke survivors [1,2] and causes various complications such as dehydration, weight loss, malnutrition [3,4], and is associated with social and psychological burden that reduce quality of life for patients, family, and caregivers [5,6,7]. In particular, aspiration is known to occur in about 19.5% to 42% of acute stroke patients [8], and aspiration pneumonia is a serious complication because it can lead to death. Therefore, dysphagia rehabilitation is not only important for safe swallowing, it can also reduce morbidity and mortality, reduce length of hospitalization, and healthcare expenditures [1,9,10,11].

The suprahyoid muscle, consisting of the geniohyoid, mylohyoid, digastric, and stylohyoid muscles, plays an essential role as primary muscle in the pharyngeal phase [12]. These muscles undergo kinematic effects such as triggering anterior-upward movement of the hyolaryngeal complex primarily through contraction during swallowing, particularly the anterior movement to the upper esophageal sphincter (UES) opening and the upward movement to the airway closure mechanism through airway closure [13,14,15]. However, weakened suprahyoid muscles due to stroke cause reduced movement of the hyoid bone during swallowing, resulting in insufficient UES opening, residual in the pyriform sinuses and vallecula, and aspiration/penetration [16,17]. Therefore, rehabilitation of the suprahyoid muscle is clinically important for safe swallowing.

Exercise-based dysphagia rehabilitation (EBDR) has been commonly used in clinical practice for the past three decades by multidisciplinary approach of specialized professionals including rehabilitation physicians, speech-language therapists, and occupational therapists [18,19]. In the past few years, many researchers have reported various methods of EBDR, such as shaker exercise (head lift exercise) [4,20], tongue strengthening exercise [12,21,22], expiratory muscle strengthening training [23,24], effortful swallowing [25,26], chin tuck against resistance exercise [27,28], forehead against resistance [29], chin-to-chest exercise [28,30], Mendelsohn [31,32], jaw opening exercise [33,34], proprioceptive neuromuscular facilitation technique [35], head extension swallowing exercise [36,37], swallowing against laryngeal restriction [38,39], and swallow exercise aid exercise [40,41] to improve swallowing function. These methods are known to induce high activation of muscles by providing loading to the oropharyngeal muscles, and consequently, contributing to the improvement of oropharyngeal swallowing function, increased hyoid bone movement and myophysiological changes. However, dysphagia rehabilitation through suprahyoid muscle strengthening is still challenging. Newer therapeutic methods are therefore needed. 

Recently, Park et al. reported the possibility of a new treatment method for dysphagia rehabilitation using kinesiology taping (KT) [42]. This method uses the elasticity and adhesion properties of KT to suppress the anterior-upward movement of the hyolaryngeal complex during spontaneous swallowing. This increases loading to the suprahyoid muscle and, consequently, subjects require more effort to overcome this movement during swallowing. Park et al. applied swallowing in healthy adults to approximately 50, 80% stretch, and non-KT conditions of KT. The higher the stretch of KT, the more significant muscle activation was found in the suprahyoid muscle. This highlighted a potential clinical rehabilitative approach for dysphagia. Furthermore, effortful swallowing against the tension of the tape causes increased pressure on the tongue during swallowing. Because several previous studies have demonstrated that effortful swallowing is effective for tongue activation and increased muscle strength through tongue push against the palate during swallowing [25,32,43]. Thus, resistance exercise using KT has an advantage of not only activating the suprahyoid muscle, but also contributing to the activation and contraction of the tongue muscle. However, the clinical application, thereof, in patients with dysphagia was lacking. Therefore, this study investigated the effect of dysphagia rehabilitation using KT on oropharyngeal muscle hypertrophy in patients with dysphagia after stroke. We hypothesized that dysphagia rehabilitation using KT would induce increased oropharyngeal muscle thickness.

## 2. Materials and Methods 

### 2.1. Subjects

This was a prospective, randomized, double-blind sham-controlled study. Thirty patients with dysphagia after stroke were enrolled in this study. Inclusion criteria: Dysphagia within 6 months of stroke onset, oropharyngeal dysphagia confirmed via a videofluoroscopic swallowing study, ability to swallow voluntarily, ability to swallow against resistance of tape, a cognitive disability score of >22 points in the Mini-Mental Status Examination (MMSE), adequate communication abilities, and tongue pressure >20 (kPa). Exclusion criteria: Secondary stroke, presence of other neurological diseases, underwent tracheostomy, unstable medical condition, skin problems associated with taping attachment, pain in the cervical spine, and main problems with the esophageal phase such as achalasia or UES opening. Ethical approval was obtained from the Dongseo University Institutional Review Board prior to the experiment (1041493-A-2020-001). The data collected during 1 April–20 July 2020 and this study was not registered in a public trials registry. 

### 2.2. Procedures

**Figure 1:**
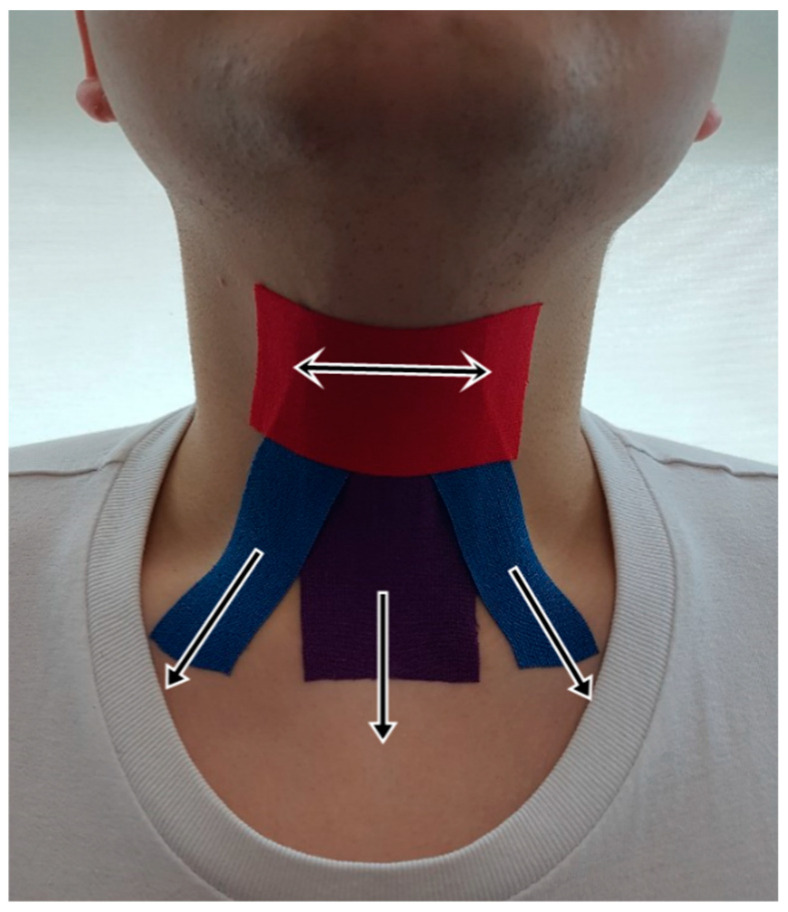
Application of the kinesiology tape.

Participants were randomly assigned to either the experimental or sham group. The method for the application of KT is as follows: Subjects sat upright in a chair, with the head and neck facing forward, and maintained a neutral position. To firmly adhere the tape, the anterior neck was wiped clean with alcohol. Three types of KT (BB Tape; WETAPE Inc., Seoul, Korea) were prepared and attached base on Park et al. [42]. Firstly, I-shaped tape was pulled downward to the level of the thyroid notch to wrap the thyroid cartilage and attach to the sternum; secondly, the reverse, V-shaped tape was attached from the hyoid bone to the medial superior surface of the clavicle; and thirdly, the hyolaryngeal complex was covered in the horizontal direction to restrict its movement during swallowing [42] (Figure 1). KT was applied at approximately 70% tension except during the third step. In the sham group, KTs were applied in the same manner as the experimental group except without the tape’s tension. 

Both groups performed repetitive swallowing exercise by applying KT, and the specific exercise protocol is as follows. After applying the KT, the subjects performed 5 sessions of repeated swallowing. Each session consisted of 10 consecutive swallows. A small amount of water was provided for smooth repeated swallowing. The subjects were given 2–3 min of rest after the completion of each session. This was done 10 times a day (a total of 50 swallows per day) for 4 weeks. 

### 2.3. Outcome Measurement

**Figure 2:**
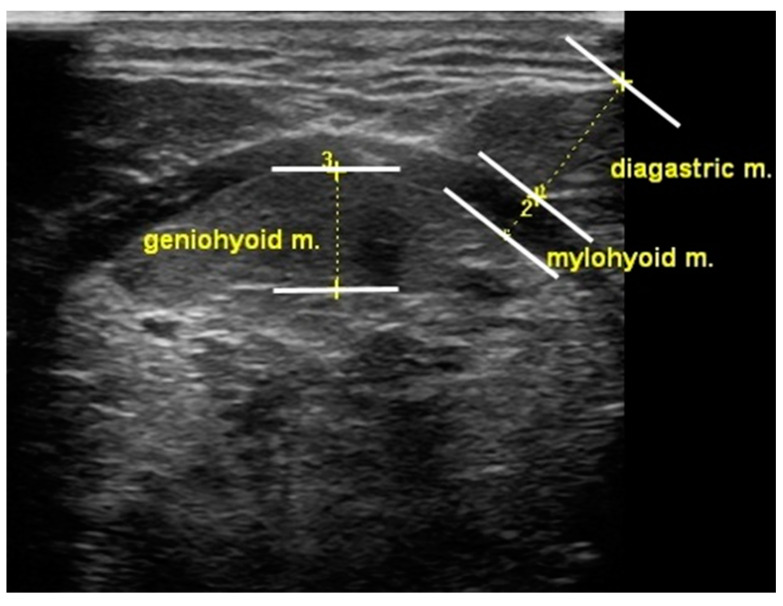
Measurement of oropharyngeal muscle thickness.

In this study, tongue and suprahyoid muscle thickness was measured using a portable ultrasonography device (SONON300L, Healcerion, Seoul, Korea) with a 10 MHz and linear- and convex-array transducer. Change in tongue thickness was determined by measuring the distance between the upper and lower surfaces of the tongue muscles in the center of the plane perpendicular to the Frankfort horizontal plane of the frontal session. The vertical distance was measured from the surface of the mylohyoid muscle to the dorsum of the tongue. The digastric muscle measurements were obtained from the upper to the lower boundary of the fascia covering the muscle at the broadest point perpendicular to the mylohyoid muscle. The mylohyoid muscle measurements were recorded below the measurement point of the digastric muscle, from the upper to the lower boundary of the fascia covering the muscle (Figure 2). A blinded investigator (rehabilitation physician) measured and analyzed the muscle thickness using ultrasound.

### 2.4. Statistical Analysis

All statistical analyses were performed using SPSS 15.0 software (SPSS Inc., Chicago, IL, USA). Descriptive statistics are presented as means with standard deviations. The Shapiro–Wilk test was used to check the normality of the outcome variables. To evaluate the effects of the intervention, the Wilcoxon signed-rank test was used to compare the pre- and postintervention measures in each group. The Mann–Whitney U test was used to compare the intergroup changes in the outcome measures. A *p*-value of <0.05 was deemed statistically significant. 

## 3. Results

### 3.1. Participants

**Figure 3:**
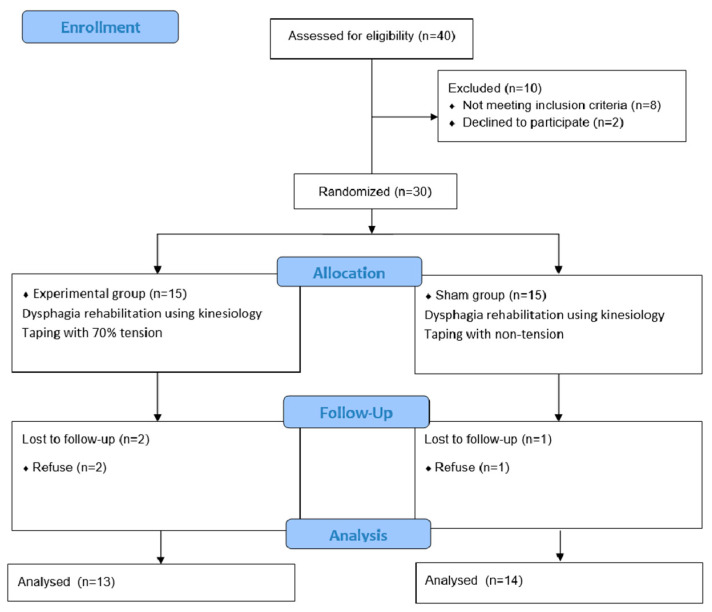
CONSORT diagram of participant recruitment.

Three study participants withdrew. The reasons for withdrawal were as follows: Two participants from the experimental groups began experiencing muscle fatigue and discomfort, and, one from the sham group was transferred to another hospital for personal reasons. Table 1 shows the general characteristics of the subjects. Before the intervention, there was no significant difference in the oropharyngeal muscle thickness between the two groups. Figure 3 shows the Consolidated Standards of Reporting Trials (CONSORT) diagram of participant recruitment.

### 3.2. Tongue Muscle Thickness

Comparison results within the group, experimental group showed a significant increase in tongue muscle thickness. Whereas, sham group showed a no significant increase in tongue muscle thickness. As a result of comparison between groups after intervention, the experimental group showed a significant increase in tongue muscle thickness than the control group (Table 2). As a result of comparing the changes in tongue muscle thickness, the experimental group also showed a significant increase compared to the sham group. The effect sizes were as follows: Tongue muscle (0.6) (Table 3). 

### 3.3. Suprahyoid Muscle Thickness

Comparison results within the group, experimental group showed a significant increase in mylohyoid muscle and the anterior belly of the digastric muscle thickness. Whereas, sham group showed a no significant increase in mylohyoid muscle and the anterior belly of the digastric muscle thickness. As a result of comparison between groups after intervention, the experimental group showed a significant increase in mylohyoid muscle and the anterior belly of the digastric muscle thickness than the control group (Table 2). As a result of comparing the changes in mylohyoid muscle and the anterior belly of the digastric muscle thickness, the experimental group also showed a significant increase compared to the sham group. The effect sizes were as follows: mylohyoid muscle (MHM) (1.1), and anterior belly of the digastric muscle (ADM) (1.0) (Table 3).

### 3.4. Side Effects

Two of the participants in the experimental group reported muscle fatigue, temporary pain, and discomfort. This eventually led to them withdrawing from the study.

## 4. Discussion

The dysphagia rehabilitation method using KT is a recently reported exercise method and the possibility of this method being effective was first proposed by Park et al. [42]. However, the clinical evidence is still insufficient and the effect of this method on patients with dysphagia is unknown. Therefore, this study was first attempted to investigate the effect of dysphagia rehabilitation for 6 weeks with KT on tongue and suprahyoid muscle thickness in patients with dysphagia after stroke. After dysphagia rehabilitation using KT for 6 weeks, the experimental group showed significantly increased thickness in the tongue muscle, MM, and ADM and a moderately large effect than the sham group. This study demonstrated that KT could be an effective rehabilitative method for dysphagia in patients post stroke.

First, the main reason for this result is well explained by the myophysiological effect of skeletal muscle through resistance exercise. This study used the feature of elasticity of the tape to provide loading to the swallowing muscle during swallowing. Patients require more effort to counter the elasticity of the tape during swallowing, which results in high muscle activation as a result of loading into the tongue and suprahyoid muscle. That is, the resistance generated from the tape causes the patients to perform effortful swallowing to overcome the resistance, and such effortful swallowing is known to be effective in activating and strengthening the tongue and suprahyoid muscle [25,32,43]. The increased muscle activity in surface electromyography (sEMG) leads to an increased motor unit activity in the peripheral nervous system [44]. This indicates that the discharge rate in motor units was increased, or the number of recruited motor units was increased [23,24]. Therefore, it is possible that repeated performance may cause muscle physiological changes such as muscle strength or thickness increase, and previous studies have also reported that resistance exercise, which can induce high muscle activity, contributes to the improvement of muscle strength and muscle thickness of the skeletal muscle [12,21,22,28]. The results of these previous studies support the results of the present study.

Resistance exercise is a good option to induce skeletal muscle thickness changes [45], but there are some considerations such as the intensity of resistance and the duration of intervention. The strength of resistance is an important factor for effective resistance exercise of skeletal muscle [46]. Generally, the strength of the resistor is set based on one repetition maximum (1 RM), and a strength of about 60%–80% of 1 RM is recommended [47,48]. However, in this study, since the resistance strength of the tape cannot be set based on 1 RM of the suprahyoid muscle during swallowing, about 70% tension of KT was applied based on the results of previous studies [42] and pilot studies of this study. Because, in this study, we piloted 10 patients using various tensions to determine the tension of KT. As a result, most patients were unable to swallow the 80% tension applied in the previous study against tape resistance. Because the previous study probably applied to healthy adults, they thought it was possible to overcome the 80% tension and spontaneously swallow. 

There are some limitations to this study. Firstly, KT tension recommended in previous studies with healthy adults was 60%–80%. It was found that the most suitable tension level for KT application in this study group was approximately 70%. However, determining the optimum tension of the tape is dependent on patient factors such as the severity of dysphagia and patient’s disease status. This process also needs validation. Secondly, the small sample size limits the study. A larger sample size with greater variable control will be able to validate the results obtained. Thirdly, the effect on the swallowing function was not ascertained because only the thickness change of the suprahyoid muscle in this study was measured. The effect of KT on swallowing function can be the primary focus of further research in dysphagia post stroke.

## 5. Conclusions

We found evidence that dysphagia rehabilitation using KT can increase the thickness of the oropharyngeal muscles. Therefore, KT may be considered a possible therapeutic approach and further studies are warranted.

## Figures and Tables

**Table 1:** General characteristics of the subjects.

	Experimental Group	Sham Group
Number of subjects	13	14
Gender (male/female)	5/8	6/8
Age (year)	71.3 ± 6.5	70.5 ± 8.2
Stroke onset (weeks)	16.2 ± 5.2	15.1 ± 6.4
Stroke type		
Hemorrhage	7	5
Infarction	6	9
Site of stroke lesion		
Middle cerebral artery	12	13
Pontine	1	1
Paretic side (Left/Right)	6/7	8/5
Taping tension	70% tension	Non-tension

**Table 2:** Comparison of results between two group.

	Experimental Group			Sham Group			Intergroup *p*-Values
	Pre-Intervention	Post-Intervention	*p*-Value in Group	Pre-Intervention	Post-Intervention	*p*-Value in Group	
TM	41.09 ± 3.19	42.86 ± 2.86	<0.001 *	39.64 ± 2.50	39.80 ± 2.53	0.182	0.007 ^†^
MHM	0.76 ± 0.11	0.88 ± 0.07	<0.001 *	0.73 ± 0.08	0.75 ± 0.10	0.231	0.002 ^†^
ADM	6.34 ± 0.36	6.67 ± 0.38	<0.001 *	6.09 ± 0.22	6.10 ± 0.21	0.198	0.001 ^†^

TM, tongue muscle; MHM, mylohyoid muscle; and ADM, anterior belly of the digastric muscle. Unit: mm, mean ± standard deviation. * *p* < 0.05 by Wilcoxon signed-rank, ^†^ *p* < 0.05 by Mann-Whitney *U* test.

**Table 3:** Comparison of improvement after treatment in each group.

	Experimental Group	Sham Group	*p*-Value
TM	1.77 ± 1.03	0.15 ± 0.35	<0.001 ^†^
MHM	0.11 ± 0.08	0.01 ± 0.04	<0.001 ^†^
ADM	0.34 ± 0.21	0.01 ± 0.03	<0.001 ^†^

TM, tongue muscle; MHM, mylohyoid muscle; and ADM, anterior belly of the digastric muscle. Unit: mm, mean ± standard deviation. ^†^ *p* < 0.05 by Mann–Whitney *U* test.
